# Innovating Care for Postmenopausal Women Using a Digital Approach for Pelvic Floor Dysfunctions: Prospective Longitudinal Cohort Study

**DOI:** 10.2196/68242

**Published:** 2025-04-02

**Authors:** Ana P Pereira, Dora Janela, Anabela C Areias, Maria Molinos, Xin Tong, Virgílio Bento, Vijay Yanamadala, Jennesa Atherton, Fernando Dias Correia, Fabíola Costa

**Affiliations:** ^1^Sword Health, Inc, Draper, UT, United States; 2 Department of Psychology, University of Virginia, Charlottesville, VA, United States; 3 Department of Surgery, Frank H Netter School of Medicine, Quinnipiac University, Hamden, CT, United States; 4 Department of Neurosurgery, Hartford Healthcare Medical Group, Westport, CT, United States; 5 Neurology Department, Centro Hospitalar e Universitário do Porto, Porto, Portugal

**Keywords:** women's health, pelvic floor muscle training, physical therapy, menopause, digital therapeutics, biofeedback, mobile phone

## Abstract

**Background:**

The menopause transition is a significant life milestone that impacts quality of life and work performance. Among menopause-related conditions, pelvic floor dysfunctions (PFDs) affect ∼40%‐50% of postmenopausal women, including urinary or fecal incontinence, genito-pelvic pain, and pelvic organ prolapse. While pelvic floor muscle training (PFMT) is the primary treatment, access barriers leave many untreated, advocating for new care delivery models.

**Objective:**

This study aims to assess the outcomes of a digital pelvic program, combining PFMT and education, in postmenopausal women with PFDs.

**Methods:**

This prospective, longitudinal study evaluated engagement, safety, and clinical outcomes of a remote digital pelvic program among postmenopausal women (n=3051) with PFDs. Education and real-time biofeedback PFMT sessions were delivered through a mobile app. The intervention was asynchronously monitored and tailored by a physical therapist specializing in pelvic health. Clinical measures assessed pelvic floor symptoms and their impact on daily life (Pelvic Floor Impact Questionnaire–short form 7, Urinary Impact Questionnaire–short form 7, Colorectal-Anal Impact Questionnaire–short form 7, and Pelvic Organ Prolapse Impact Questionnaire–short form 7), mental health, and work productivity and activity impairment. Structural equation modeling and minimal clinically important change response rates were used for analysis.

**Results:**

The digital pelvic program had a high completion rate of 77.6% (2367/3051), as well as a high engagement and satisfaction level (8.6 out of 10). The safety of the intervention was supported by the low number of adverse events reported (21/3051, 0.69%). The overall impact of pelvic floor symptoms in participants’ daily lives decreased significantly (−19.55 points, 95% CI −22.22 to −16.88; *P<*.001; response rate of 59.5%, 95% CI 54.9%-63.9%), regardless of condition. Notably, nonwork-related activities and productivity impairment were reduced by around half at the intervention-end (−18.09, 95% CI −19.99 to −16.20 and −15.08, 95% CI −17.52 to −12.64, respectively; *P*<.001). Mental health also improved, with 76.1% (95% CI 60.7%-84.9%; unadjusted: 97/149, 65.1%) and 54.1% (95% CI 39%-68.5%; unadjusted: 70/155, 45.2%) of participants with moderate to severe symptomatology achieving the minimal clinically important change for anxiety and depression, respectively. Recovery was generally not influenced by the higher baseline symptoms’ burden in individuals with younger age, high BMI, social deprivation, and residence in urban areas, except for pelvic health symptoms where lower BMI levels (*P*=.02) and higher social deprivation (*P*=.04) were associated with a steeper recovery.

**Conclusions:**

This study demonstrates the feasibility, safety, and positive clinical outcomes of a fully remote digital pelvic program to significantly improve PFD symptoms, mental health, and work productivity in postmenopausal women while enhancing equitable access to personalized interventions that empower women to manage their condition and improve their quality of life.

## Introduction

The menopause transition is a major health milestone women face, often associated with a significant psychological, behavioral, and social impact, that can deeply decrease productivity and overall quality of life [1-4]. Every year, about 27 million women in the United States workforce experience menopause, corresponding to approximately 20% of the workforce [5]. Menopause-related symptoms, ranging from cognitive or mental to pelvic floor dysfunctions (PFDs), often emerge between the ages of 44 and 56 years (average age of 51 years in the United States) [1,3], just when women are likely to move into leadership positions [5,6]. Menopause has therefore been pointed as a risk factor for female talent loss [5-7]. Notably, 1 in 5 women have either quit or considered leaving their job due to the severity of menopause symptoms [5,6]. Overall costs attributable to menopause symptoms were estimated to amount to up to US $26 billion per year in the United States alone, including US $1.8 billion stemming from indirect costs associated with impaired work productivity [6].

Among menopause-related symptoms, PFDs are particularly prevalent, affecting ∼40%‐50% of postmenopausal women [8,9]. Due to the decline in estrogen, the pelvic floor muscles and tissues become thinner, drier, less elastic, and weaker, which can result in conditions such as urinary or fecal incontinence, genito-pelvic pain, and pelvic organ prolapse [10-14].

First-line interventions for PFDs encompass pelvic floor muscle training (PFMT), education, and behavioral change strategies [15-21]. PFMT has proven effectiveness in the management of urinary incontinence [22-28], bowel symptoms [26], pelvic organ prolapse [24,26], and genito-pelvic pain [29,30] in postmenopausal women.

Despite high symptom prevalence and their negative impact, many women do not seek care at all (up to 61%) [31-33]. Low treatment rates are related to multiple barriers to care, including geographical and time constraints, persistent social stigma, the perception that symptoms are a “normal” part of aging [31-33], and lack of access to trained providers [34-36] or awareness of available treatment options [31-33]. Consequently, the disease burden experienced by these women might increase. As an example, in a randomized controlled trial, postmenopausal women (n=48) who did not receive PFMT experienced a 50% increase in urinary leakage after one year, while those who performed PFMT showed significant improvement in their condition [27], underscoring the need for appropriate care for this population.

Digital pelvic rehabilitation solutions have arisen as a potential alternative to improve both access and adherence to treatment. Digital interventions have already been reported to be effective against urinary incontinence [37,38], even when compared to in-person PFMT [39]. Additionally, in a study including mostly postmenopausal women, a self-management mobile app for urinary incontinence has shown higher improvement in quality of life and urinary-related symptoms than an information-only intervention [40]. However, further evidence is lacking on the specific needs of postmenopausal women, including those with PFDs other than urinary incontinence.

This study aims to assess the feasibility, engagement, and clinical outcomes (namely the impact of PFDs on physical, mental, and work productivity) of postmenopausal women after a fully remote digital pelvic program, combining education with PFMT using real-time biofeedback. This digital pelvic program is asynchronously managed by a physical therapist (PT), and it has previously shown significant clinical improvements in managing urinary incontinence in women [41]. This study hypothesizes that participants would report improvement in all outcomes after the program. The findings collected could inform health care providers, researchers, and policy makers about the potential of remote digital pelvic programs to improve health outcomes and accessibility for postmenopausal women with PFDs.

## Methods

### Study Design

This is a real-world, prospective single-arm observational cohort study. The recruitment period was from December 1, 2022, until July 29, 2024, and the home-based digital pelvic program was conducted between December 1, 2022, and August 8, 2024. This study was reported in accordance with STROBE (Strengthening the Reporting of Observational Studies in Epidemiology) guidelines (Table S1 in Multimedia Appendix 1).

### Ethical Considerations

This research was conducted in accordance with the Declaration of Helsinki and all applicable ethical guidelines and regulations. The protocol was prospectively approved by the Advarra Institutional Review Board (Pro00064510) and registered on ClinicalTrials.gov (NCT05513417) on August 24, 2022. Electronic informed consent was obtained from all participants. Participants were informed of their right to withdraw from the study at any time without any adverse consequences. All collected data underwent a rigorous anonymization process to safeguard the privacy of the individuals involved in the research. The data collection and analysis procedures complied with established guidelines and regulations. Participants were not offered any form of compensation.

### Participants

Female beneficiaries of employers or health plans covered by the Sword Bloom program (Draper, Utah, United States) across 50 states in the United States and the District of Columbia were invited to apply. Individuals in the postmenopausal phase, defined as at least 1 year after their final menstrual period [42], who reported PFDs encompassing conditions such as urinary conditions (including incontinence and voiding dysfunctions), pelvic organ prolapse, bowel conditions, and genito-pelvic pain were included in this study. This diverse array of PFDs was included considering common conditions among postmenopausal women [3,14,43], and to evaluate the feasibility and outcomes of the intervention across multiple conditions for which pelvic rehabilitation is recommended as first-line treatment [15-21,30,44].

Exclusion criteria comprised: (1) inability to perform 20 minutes of light to moderate exercise; (2) active cancer or under treatment for cancer; (3) surgery, significant trauma, or other conditions where mobilization is contraindicated; (4) clinical red flags suggestive of serious underlying conditions not cleared by their attending physician; (5) signs of acute, serious neurologic compromise; (6) clinical conditions (eg, dementia) precluding compliance with autonomous home-based exercise; (7) pelvic infection or suspicion of inflammatory bowel disease; (8) contraindication for the use of an intravaginal device; and (9) allergy to silicone. These criteria were selected in order to exclude participants that require medical referral and conditions that prevent autonomous engagement with the program, guaranteeing participant’s safety.

Participants were considered dropouts in the absence of exercise sessions for 30 consecutive days. Those who did not complete reassessment surveys but were compliant with the intervention were not excluded.

### Intervention

The intervention had an average duration of 10 weeks and consisted of biofeedback-mediated PFMT, functional exercises, and education (Table S2 in Multimedia Appendix 1), as previously described [41]. Briefly, after enrollment, participants completed an onboarding form with demographic and clinical information and selected a PT specializing in pelvic health who oversaw and continuously tailored the treatment. During the onboarding video call, the PT conducted the initial clinical evaluation, leveraging the information provided by the participant upon enrollment. During anamnesis, PTs confirmed the absence of red flags indicating potential serious conditions requiring medical screening and assessed the participant’s clinical presentation (including the participant’s ability to contract the PFM and the presence of comorbid pelvic floor conditions). Additionally, PTs provided education (about PFDs, PFMT, diaphragmatic breathing training, behavioral modifications, and relevant information regarding the use and maintenance of the biofeedback device), and established the intervention goals collaboratively with the patient, based on the clinical practice guidelines [15-21,30,44].

**Figure 1.**
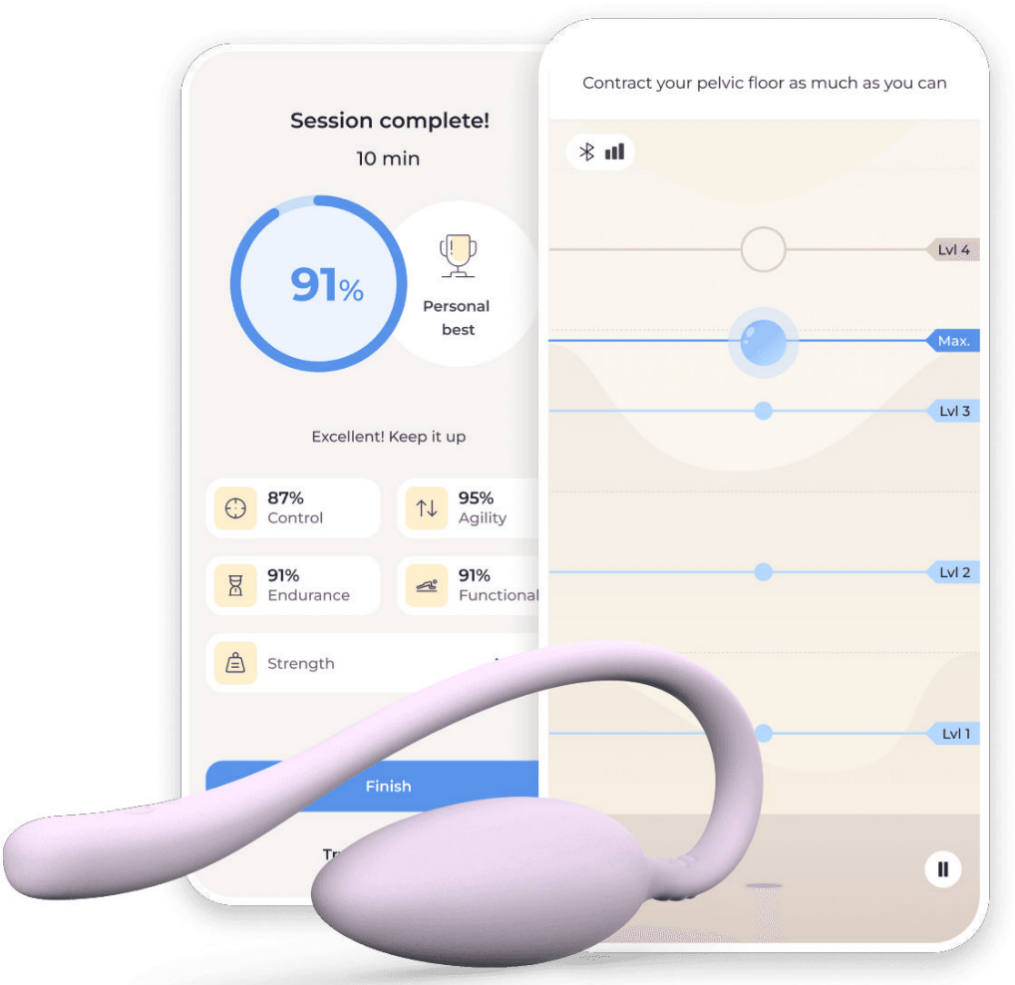
Representation of the intravaginal pod and interventional mobile app screenshots, featuring an exercise session.

This digital pelvic program used a Food and Drug Administration–listed medical device composed of an intravaginal sensor (shipped to each participant), a dedicated mobile app (to be downloaded to the participant’s smartphone), and a cloud-based platform, allowing remote monitoring and care (Figure 1). The intravaginal sensor included a force transducer to monitor pelvic floor muscle activity (contraction and relaxation) and an accelerometer to assess pelvic floor muscle motion. The data captured by the intravaginal sensor was accessed remotely by the PT through a cloud-based portal, allowing the asynchronous assessment and monitoring of the pelvic floor muscles function in different aspects (eg, maximal strength and endurance).

Gamified exercise sessions (4 sessions per week as the default recommendation) were displayed in the app, accompanied by written instructions, with the intravaginal sensor providing real-time biofeedback based on the prescribed contraction and relaxation targets.

The educational content on pelvic health followed current clinical guidelines and research [15,16,44] and included the role of mental health and topics tailored to each specific PFD (eg, bladder retraining and toilet habits). These were selected by the attending PT and were delivered in the form of written studies and videos via the app throughout the digital pelvic program. Both exercise performance data and education content engagement were stored on a cloud-based platform, being continuously monitored by the PT to asynchronously assess progress and adjust the treatment plan.

### Outcomes

Outcomes included engagement, satisfaction, adverse events, and clinical outcomes, which are described in Table 1. Clinical outcomes assessment surveys were completed at baseline, session 9, session 15, and session 21 as long as participants reached that milestone.

**Table 1.** Study engagement, satisfaction, and clinical outcomes.

Outcome and measure	Description
Engagement metrics	
Completion rates	Percentage of participants completing the program
Sessions	Number of total sessions performed
Education	Number of educational pieces read or watched
Interaction	Number of text interactions exchanged between participant and PT^a^
Program satisfaction	
Satisfaction	Through the question “On a scale from 0 to 10, how likely are you to recommend Bloom’s programs to a friend or family member?”; range: 0‐10 (higher scores indicate higher satisfaction)
Safety	
Adverse events rate	Self-reported adverse events
Clinical outcomes	
Pelvic floor symptoms	Pelvic Floor Impact Questionnaire–short form 7 [45]; range: 0‐300 (higher scores indicate greater impact). MCIC^b^ threshold: 12% [45] Urinary Impact Questionnaire–short form 7 [44-46]; range: 0‐100 (higher scores indicate greater impact) Colorectal-Anal Impact Questionnaire–short form 7 [45]; range: 0‐100 (higher scores indicate greater impact) Pelvic Organ Prolapse Impact Questionnaire–short form 7 [45]; range: 0‐100 (higher scores indicate greater impact) Through the question “In the past 7 days, how would you rate the severity of your pelvic health symptoms?”; range: 0‐10 (higher scores indicate greater severity)
Mental health	Depression through Patient Health 9-item Questionnaire [47]; range: 0‐27 (higher scores indicate greater symptoms). MCIC: ≥5 [48] Anxiety through Generalized Anxiety Disorder 7-item scale [49]; range: 0‐21 (higher scores indicate greater symptoms). MCIC: ≥3.8 [50]
WPAI^c^	WPAI questionnaire–General Health v2.0 [51]; range: 0%‐100% (higher scores indicate greater impairment)

a PT: physical therapist.

b MCIC: minimal clinically important change.

c WPAI: work productivity and activity impairment.

### Safety and Adverse Events

Participants were asked to report any adverse events to their assigned PT. An adverse event was defined as any undesirable experience associated with the use of a medical product by a participant [52]. An adverse event was considered serious if it was life-threatening, required hospitalization, or led to disability, permanent damage, congenital anomaly, or birth defect. Reported events were classified as related, not related, or of unknown relationship to the intervention. Regular and on-demand contact between participants and PTs was maintained via a secure in-app chat for support, feedback, motivation, and safety assurance. Additionally, symptoms and fatigue levels (rated on a 0‐10 scale) were also self-reported after each exercise session and remotely monitored by the PT.

### Statistical Analysis

A latent growth curve analysis (LGCA) was used to model clinical outcome trajectories across sessions following an intention-to-treat approach. LGCA is a type of structural equation model that calculates overall change based on individual trajectories, considering time as continuous [53]. Advantages of this methodology include the provision of model fit measures, and the handling of missing data through full information maximum likelihood, which outperforms listwise deletion and other imputation models [54,55]. A robust sandwich estimator was used for standard errors. A conditional LGCA was conducted to assess the influence of covariates—age, BMI, hormone replacement therapy (yes or no), social deprivation index [56], and rurality (rural or urban)—on intercept, slope, and curve, fitted as random effects.

Latent-basis growth analysis (LBGA), following an intention-to-treat approach, was conducted for Work Productivity and Activity Impairment Questionnaire (WPAI) and Pelvic Health Symptoms outcomes, as LGCA did not result in a satisfactory model fit. LBGA provided a better model fit as it accounts for variations in the pace and timing of change across individuals [57]. These analyses considered those with baseline scores of >0. A conditional LBGA was also performed with the same mentioned covariates.

Response rates for Pelvic Floor Impact Questionnaire–short form-7 (PFIQ-7), Patient Health 9-item Questionnaire (PHQ-9), and Generalized Anxiety Disorder 7-item (GAD-7) were calculated for participants who completed the program and reported baseline clinically relevant scores (ie, PHQ-9 ≥10 and GAD-7 ≥10, respectively), using the respective minimal clinically important change (MCIC) thresholds (Table 1). These response rates were adjusted for the aforementioned covariates.

Statistical analyses were performed using R Studio (version 2023.09.1+494; Posit, PBC). Statistical significance was defined as *P<*.05 considering a 2-sided hypothesis test.

## Results

### Overview

**Figure 2.**
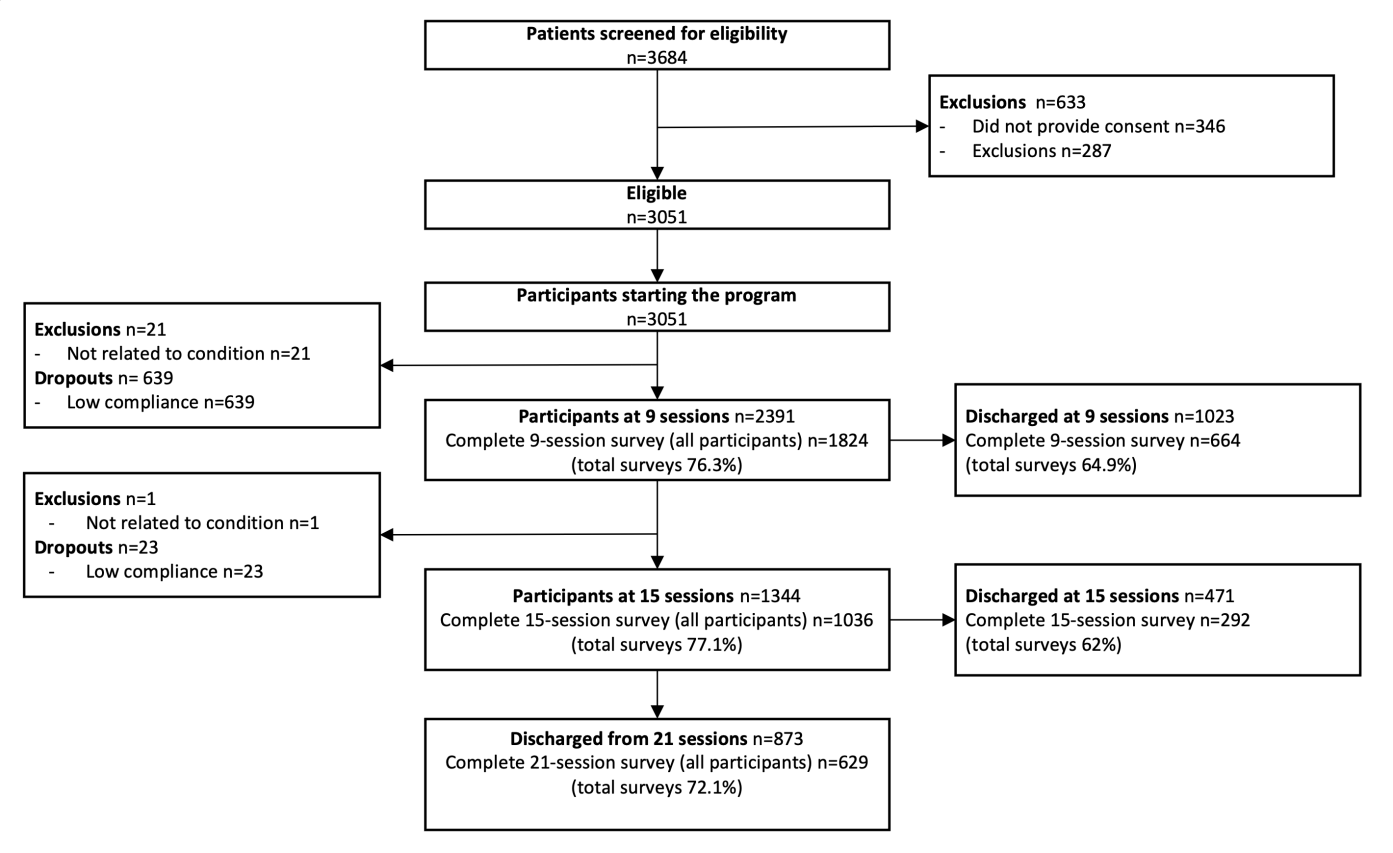
Study flowchart.

Of the 3684 participants screened for eligibility, 633 participants were excluded (346 declined participation and 287 did not meet eligibility criteria; Figure 2). The program started with 3051 participants, of which 2367 participants completed it, translating into a completion rate of 77.6%.

### Baseline Characteristics

Overall, the cohort was on average 54.3 (SD 6.5) years old, with an average BMI of 29.4 (SD 6.8) kg/m^2^, having a high proportion of participants with obesity (1197/3051, 39.2%), of those with higher education levels (1826/3051, 59.9%) and full-time employed (2332/3051, 76.4%). Regarding races or ethnicities, 18.5% (563/3051) were from minority groups (Table 2). Of those who reported parity, 87.1% (1763/2025) were parous or multiparous (Table 2). Urinary conditions were the most common PFD (2174/3051, 71.3%), followed by genito-pelvic pain (597/3051, 19.6%; Table 2).

**Table 2.** Baseline characteristics of study participants: entire cohort (n=3051).

Characteristic	Entire cohort (n=3051)
Age (years), mean (SD)	54.3 (6.5)
Age categories (years), n (%)	
35‐40	45 (1.5)
41‐54	1595 (52.3)
≥55	1411 (46.2)
Gender, n (%)	
Women	2879 (94.4)
Nonbinary	12 (0.4)
Prefer not so to specify or not available	160 (5.2)
BMI (kg/m^2^; n=3038), mean (SD)	29.4 (6.8)
BMI categories (kg/m^2^; n=3038), n (%)	
Underweight (<18.5)	24 (0.8)
Normal (18.5‐25)	879 (28.9)
Overweight (≥25‐30)	938 (30.9)
Obesity (≥30‐40)	971 (32)
Severe obesity (>40)	226 (7.4)
Employment status, n (%)	
Employed full-time	2332 (76.4)
Employed part-time	253 (8.3)
Unemployed	211 (6.9)
Retired	198 (6.5)
Prefer not to specify	57 (1.9)
Education level, n (%)	
Less than high school	11 (0.4)
High school diploma	273 (8.9)
Some college	903 (29.6)
Bachelor’s degree	1149 (37.7)
Graduate degree	677 (22.2)
Prefer not to specify	38 (1.2)
Race or ethnicity, n (%)	
American Indian or Alaska Native	9 (0.3)
Asian or Pacific Islander	73 (2.4)
Black or African American	219 (7.2)
Hispanic or Latino	255 (8.4)
Multiracial or biracial	43 (1.4)
Not listed	7 (0.2)
Prefer not to specify	47 (1.5)
White or Caucasian	2398 (78.6)
Geographic location, n (%)	
Rural	437 (14.3)
Urban	2608 (85.5)
Not available	6 (0.2)
Social Deprivation Index (n=3045), n (%):	
1‐20 (less deprived)	1025 (33.6)
21‐40	728 (23.9)
41‐60	576 (18.9)
61‐80	428 (14)
81‐100 (more deprived)	288 (9.4)
Pelvic floor dysfunction, n (%)	
Genito-pelvic pain or penetration disorder	597 (19.6)
Bowel conditions	58 (1.9)
Pelvic organ prolapse	198 (6.5)
Urinary conditions	2174 (71.3)
Multiple conditions	24 (0.8)
Parity, n (%)	
Nulliparous	262 (8.5)
Parous or multiparous	1763 (57.8)
Not available	1026 (33.6)
Self-reported use of hormone replacement therapy (n=260), n (%)	260 (8.5)
Clinical outcomes, mean (SD)	
PFIQ-7^a^	49.5 (51.9)
UIQ-7^b^ (n=3043)	24.7 (22.8)
CRAIQ-7^c^ (n=2887)	11.3 (19.8)
POPIQ-7^d^ (n=2981)	14.5 (20.6)
Pelvic Health Symptoms >0 (n=2855)	4.81 (2.01)
GAD-7^e^	3.59 (4.4)
GAD-7 ≥10 (n=289)	13.8 (3.4)
PHQ-9^f^	2.64 (4.6)
PHQ ≥10 (n=297)	13.8 (3.6)
WPAI^g^ Overall >0 (n=1048)	31.2 (22.6)
WPAI Work >0 (n=990)	28.1 (19.3)
WPAI Activities >0 (n=1571)	33.2 (22.3)

a PFIQ-7: Pelvic Floor Impact Questionnaire–short form 7.

b UIQ-7: Urinary Impact Questionnaire–short form 7.

c CRAIQ-7: Colo-Rectal-Anal Impact Questionnaire–short form 7.

d POPIQ-7: Pelvic Organ Prolapse Impact Questionnaire–short form 7.

e GAD-7: Generalized Anxiety Disorder 7-item scale.

f PHQ-9: Patient Health 9-item Questionnaire.

g WPAI: Work Productivity and Activity Impairment Questionnaire.

### Engagement Outcomes

Treatment duration lasted 9.9 (SD 8.98) weeks on average, with participants completing a mean of 19.1 (SD 16.2) PFMT biofeedback-assisted sessions. Participants also demonstrated high engagement with the educational component, consulting on average 14.1 (SD 18.4) educational materials. Communication between participants and PTs was frequent throughout the program, amounting to a mean of 22.1 (SD 9.9) text interactions. Satisfaction with the program was 8.6 (SD 2.0) out of 10.

### Safety

Throughout the intervention, a total of 21 (out of 3051, 0.69%) adverse events were reported. Of these, 1 vaginal abrasion was reported, 1 case of vaginal bleeding that was determined to be unrelated to the intervention, and 19 cases of yeast infection or urinary tract infections were reported with unknown relation to the intervention. No serious adverse events were reported.

### Clinical Outcomes

Model estimates derived from longitudinal analyses are presented in Table 3, showing a good model fit (Table S3 in Multimedia Appendix 1). Estimates from the conditional models are described in Table S4 in Multimedia Appendix 1 and the respective model fit in Table S5 in Multimedia Appendix 1.

**Table 3.** Model estimates of clinical outcome measures following an unconditional intent-to-treat approach.

Outcome		Baseline, mean (95% CI)	Program-end, mean (95% CI)	Change, mean (95% CI)	*P* value
PFIQ-7^a^ (n=3051)		49.59 (47.75 to 51.43)	30.04 (27.45 to 32.63)	−19.55 (−22.22 to −16.88)	<.001
UIQ-7^b^ (n=3043)		24.70 (23.90 to 25.51)	14.35 (13.33 to 15.36)	−10.36 (−11.41 to −9.30)	<.001
CRAIQ-7^c^ (n=2887)		11.33 (10.60 to 12.05)	7.57 (6.54 to 8.59)	−3.76 (−4.83 to −2.69)	<.001
POPIQ-7^d^ (n=2981)		14.58 (13.85 to 15.32)	8.96 (7.92 to 10.00)	−5.62 (−6.73 to −4.51)	<.001
Pelvic Health Symptoms >0 (n=2855)		4.81 (4.74 to 4.88)	2.84 (2.70 to 2.98)	−1.97 (−2.12 to −1.83)	<.001
WPAI^e^ Overall >0 (n=1048)		31.21 (29.84 to 32.58)	16.13 (13.78 to 18.48)	−15.08 (−17.52 to −12.64)	<.001
WPAI Work >0 (n=990)		28.13 (26.93 to 29.33)	13.73 (11.69 to 15.78)	−14.40 (−16.57 to −12.22)	<.001
WPAI Activities >0 (n=1571)		33.14 (32.04 to 34.24)	15.05 (13.23 to 16.86)	−18.09 (−19.99 to −16.20)	<.001

a PFIQ-7: Pelvic Floor Impact Questionnaire–short form 7.

b UIQ-7: Urinary Impact Questionnaire–short form 7.

c CRAIQ-7: Colo-Rectal-Anal Impact Questionnaire–short form 7.

d POPIQ-7: Pelvic Organ Prolapse Impact Questionnaire–short form 7.

e WPAI: Work Productivity and Activity Impairment Questionnaire.

### Pelvic Floor Symptoms

The impact of pelvic floor symptoms in participants’ daily activities was significantly decreased by the program-end. Specifically, PFIQ-7 scores decreased by 19.55 points (95% CI −22.22 to −16.88; *P<.*001; Table 3) from a baseline score of 49.59 points (95% CI 47.75-51.43; *P<*.001), resulting in a treatment response rate of 59.5% (95% CI 54.9%-63.9%; unadjusted: 1125/1843, 61%).

Similarly, significant improvements were observed in bladder symptoms (Urinary Impact Questionnaire–short form 7 [UIQ-7]), with a reduction of 10.36 (95% CI −11.41 to −9.30; *P<.*001), as well as in bowel symptoms (Colorectal-Anal Impact Questionnaire–short form 7 [CRAIQ-7]: −3.76, 95% CI −4.83 to −2.69; *P<*.001), pelvic organ prolapse symptoms (Pelvic Organ Prolapse Impact Questionnaire–short form 7 [POPIQ-7]: −5.62, 95% CI −6.73 to −4.51; *P<*.001), and pelvic health symptoms (−1.97, 95% CI −2.12 to −1.83).

Additionally, a change of 18.09 points (95% CI −19.99 to −16.20; *P<*.001) was also observed in WPAI daily activities, reflecting a 54.6% (18.09/33.14) overall improvement in the cohort’s ability to perform nonwork-related activities.

### Mental Health

At baseline, 9.5% (289/3051) of participants reported moderate-to-severe anxiety symptoms (GAD-7 ≥10), whereas 9.7% (297/3051) participants reported moderate-to-severe depression symptoms (PHQ-9 ≥10). Mental distress was significantly reduced at program-end among these participants, with 76.1% (95% CI 60.7%-84.9%) reaching the MCIC for anxiety and 54.1% (95% CI 39%-68.5%) reaching the MCIC for depression (unadjusted: 97/149, 65.1% for anxiety and 70/155, 45.2% for depression).

### Work Productivity

Work productivity impairment had a mean score of 31.21 (95% CI 29.84-32.58) at baseline, which decreased to nearly half at intervention-end (mean change: −15.08, 95% CI −17.52 to −12.64; *P<*.001), representing a 48.3% (15.08/31.21) change.

### Impact of Covariates

At baseline, higher social deprivation was associated with a greater pelvic floor symptoms severity (*P<.*001) and impact on daily living (PFIQ-7; *P<.*001), including both bladder (UIQ-7; *P<.*001), bowel (CRAIQ-7; *P=.*001), and pelvic organ prolapse (POPIQ-7; *P=.*004 and *P=.*009, respectively; Table S4 in Multimedia Appendix 1). Additionally, participants with older age and higher BMI also reported more severe pelvic floor symptoms (*P<.*001) and higher symptoms impact on daily living (PFIQ-7, UIQ-7, CRAIQ-7, and POPIQ-7; all *P=.*001; Table S4 in Multimedia Appendix 1), respectively. Those living with higher BMI also reported a greater impairment in daily activities (WPAI activities; *P*=.003). A lower impairment in nonwork-related activities was observed among participants residing in rural locations (*P=.*045). Younger age was related to both greater impact of pelvic organ prolapse symptoms on daily living (*P=.*003) and impairment in nonwork-related activities (*P=.*006) at baseline.

Covariates did not affect the recovery of any of the clinical outcomes assessed (*P≥.*05; Table S4 in Multimedia Appendix 1), except for pelvic health symptoms where lower BMI levels (*P=.*02) and higher social deprivation (*P=.*04) were associated with a steeper recovery.

## Discussion

### Principal Results

This study provides real-world evidence to support the feasibility, safety, and positive clinical outcomes of a digital pelvic program for postmenopausal women. A high completion rate of 77.6% (2367/3051) was observed, alongside high engagement and satisfaction levels with the program (8.6/10, SD 2.0). Adverse events were low (21/3051, 0.69%), suggesting the safety of this digital pelvic program. Pelvic floor symptoms were significantly reduced at program-end (−1.97 points, 95% CI −2.12 to −1.83; *P<.*001), as well as their impact on daily lives (−19.55 points, 95% CI −22.22 to −16.88; *P<.*001), with a treatment adjusted response rate of 59.5% (95% CI 54.9%-63.9%; unadjusted: 1125/1843, 61%). Pelvic floor symptoms were reduced at intervention-end regardless of the specific condition—bladder, bowel, pelvic organ prolapse, or genito-pelvic symptoms (all *P<.*001). This also translated into a substantial reduction in impairment in carrying out nonwork-related activities (18.09/33.14, 54.6%; *P<.*001). Mental health outcomes among participants with moderate-to-severe anxiety or depression symptoms were improved at program-end, with 76.1% (95% CI 60.7%-84.9%; unadjusted: 97/149, 65.1%) and 54.1% (95% CI 39%-68.5%; unadjusted: 70/155, 45.2%) of participants reaching the MCIC for anxiety and depression, respectively. Work productivity impairment was decreased by nearly half at intervention-end (15.08/31.21, 48.3%; *P<.*001). Outcomes were not generally impacted by age, BMI, hormone replacement therapy, social deprivation, or rurality. This study showcases for the first time the benefit of a digital rehabilitation program, including education and biofeedback-guided PFMT, to improve physical, mental, and work productivity outcomes in postmenopausal women.

### Comparison With Prior Work

#### Engagement With the Program

Herein, we explore the potential of digital interventions in providing effective and scalable avenues that facilitate access to care, and importantly, stimulate compliance in postmenopausal women. In this study, a diverse cohort was evaluated, encompassing participants from various socioeconomic, rural, and racial or ethnic backgrounds, following the diversity observed in the US general population [58]. The program completion rate was high (2367/3051, 77%) and was accompanied by high adherence as translated by the number of biofeedback-assisted PFMT sessions performed (mean 19.1, SD 16.2) and the educational content consulted (mean 14.1, SD 18.4), reinforcing the role of remote interventions in promoting equitable access. Prior studies using PFMT for postmenopausal women have often combined in-person sessions with home-based prescriptions for unsupervised PFMT [23,26,27,59], to promote higher treatment frequency, which is not achievable through in-person settings alone. The high engagement in this digital pelvic program matches the range reported in past controlled studies [22]. The observed engagement may stem from privacy, lack of stigma, and convenience of digital care, enabling women to engage with the program at their own pace and fit sessions into their daily routines [26]. Furthermore, participants could openly discuss their condition with PTs specialized in pelvic health throughout the program as needed (mean text interactions 22.1, SD 9.9). This likely contributed to building a strong therapeutic alliance and fostering an empathetic, nonjudgmental environment, as reflected by the high satisfaction levels of 8.6 (SD 2.0) out of 10, similar to the reported previous in-person PFMT studies [60-62]. Additionally, the biofeedback feature may have also contributed to the high adherence observed. Previous studies have shown a positive impact of biofeedback on the autonomous performance of PFMT, guiding women in correctly contracting their pelvic floor muscles [63], promoting PFMT self-efficacy [64,65], higher adherence [66], and recovery likelihood (by 3 times compared to no biofeedback) [67].

#### Safety

A significant concern with any intervention is the possibility of the occurrence of side effects that could negatively impact a participant’s health. As such, the monitoring of potential adverse events is one of the mandatory requests in health care. Herein, the number of adverse events was low and no serious adverse events occurred, consistent with current literature for in-person interventions [26,27,59], suggesting the safety of this fully remote intervention.

#### Clinical and Productivity Outcomes

This digital pelvic program led to significant improvements in pelvic floor symptomatology, reflected by a high treatment response rate (59.5%, 95% CI 54.9%-63.9%; unadjusted: 1125/1843, 61%), which was observed regardless of the condition. The findings of this study are consistent with prior research showing the benefits of in-person PFMT in reducing pelvic floor symptoms and their impact on daily living among postmenopausal women [22-28,59]. Moreover, PFIQ-7 improvements were achieved regardless of the impact of age, BMI, self-reported hormone replacement therapy use, social deprivation, and rurality, which is particularly encouraging given that these factors contribute to PFD onset [68] or are typically associated with poorer outcomes after PFMT [69-71]. According to previous reports from patients with PFDs, mental distress is frequently associated with menopause, namely through feelings of discomfort, helplessness, and mood deterioration, all of which subsequently contribute to a diminished quality of life [72-76]. Herein, participants with moderate-to-severe mental distress reported significant improvements, with treatment response rates reaching 76.1% (95% CI 60.7%-84.9%; unadjusted: 97/149, 65.1%) for anxiety and 54.1% (95% CI 39%-68.5%; unadjusted: 70/155, 45.2%) for depression. These results align with a previous systematic review focused on postmenopausal women who reported significant effects of exercise on mental outcomes [77]. This underscores the importance of considering mental health in managing PFDs, as mental distress predicts poorer recovery following PFMT, and improvements in depression are linked to better PFD symptom outcomes [78].

Adding to the positive clinical outcomes observed, productivity was also significantly improved after this fully remote digital pelvic program (15.08/31.21, 48.3%; *P<.*001). The overarching impact of menopause on productivity is well-known [3,79], encompassing tremendous socioeconomic consequences and even loss of female talent [5-7]. Therefore, preliminary studies have been delving into workplace strategies to support women in dealing with menopause-related symptoms and mitigate the associated challenges [80]. A self-help cognitive behavioral therapy booklet promoted improvements in work presenteeism by 15% and in work and social adjustment (32%) [81]. Although measured by different patient-reported outcomes, our results exceed these findings, showcasing the potential of multimodal digital pelvic programs in helping women manage symptoms and thrive in their professional lives, especially when offered as an employee benefit. Overall, these results showcase, for the first time, the benefits of a digital pelvic program, including education and biofeedback-guided PFMT, to improve physical, mental, and work productivity outcomes, while providing equitable access to care, in postmenopausal women from all socioeconomic backgrounds.

### Limitations

This study presents some limitations that warrant discussion: (1) the observational study design that lacks a control group precludes the establishment of a causal effect; (2) the lack of pelvic floor muscle function outcomes, such as strength and endurance, preventing the assessment of objective measures in this domain; and (3) the concomitant use of other medication besides hormone replacement therapy by some participants (eg, anticholinergics), or other nonpharmacological interventions (eg, pessaries), which may be a source of potential confounding. Nevertheless, the use of hormone replacement therapy was collected and used as a covariate when analyzing outcomes. Future research is needed to determine the maintenance of the observed improvements through long-term follow-ups.

### Conclusions

This study demonstrates the feasibility, safety, and positive clinical outcomes, namely on pelvic floor symptoms, mental health status, and work productivity, after a fully remote, biofeedback-assisted digital pelvic program in postmenopausal women with PFDs. These findings highlight the potential of digital care to enhance equitable access to timely, scalable, and personalized pelvic health interventions. By empowering postmenopausal women to manage their pelvic health disorders more effectively, this approach enhances their overall quality of life, while leveling the opportunities to thrive in the workplace.

## Supplementary material

10.2196/68242 Multimedia Appendix 1 Supplementary tables.

## Abbreviations

CRAIQ-7: Colo-Rectal-Anal Impact Questionnaire—short form 7
GAD-7: Generalized Anxiety Disorder 7-item scale
LBGA: latent-basis growth analysis
LGCA: latent growth curve analysis
MCIC: minimal clinically important change
PFD: pelvic floor dysfunction
PFIQ-7: Pelvic Floor Impact Questionnaire—short form 7
PFMT: pelvic floor muscle training
PHQ-9: Patient Health 9-item Questionnaire
POPIQ-7: Pelvic Organ Prolapse Impact Questionnaire—short form 7
PT: physical therapist
STROBE: Strengthening the Reporting of Observational Studies in Epidemiology
UIQ-7: Urinary Impact Questionnaire—short form 7
WPAI: Work Productivity and Activity Impairment Questionnaire
