# Supplementary material for: Innovating Care for Postmenopausal Women Using a Digital Approach for Pelvic Floor Dysfunctions: Prospective Longitudinal Cohort Study
Source: JMIR Mhealth Uhealth. 2025 Apr 2;13:e68242. doi: 10.2196/68242 (PMC12038761; doi:10.2196/68242)
Supplement: Multimedia Appendix 1 [file mhealth-v13-e68242-s001.pdf]

Table S1. STROBE reporting checklist.

	Item No	Recommendation	Page No
**Title and abstract**	1	(*a*) Indicate the study's design with a commonly used term in the title or the abstract<br>(*b*) Provide in the abstract an informative and balanced summary of what was done and what was found	1
**Introduction**			
Background/rationale	2	Explain the scientific background and rationale for the investigation being reported	2
Objectives	3	State specific objectives, including any prespecified hypotheses	2
**Methods**			
Study design	4	Present key elements of study design early in the paper	2
Setting	5	Describe the setting, locations, and relevant dates, including periods of recruitment, exposure, follow-up, and data collection	2-3
Participants	6	(*a*) Give the eligibility criteria, and the sources and methods of selection of participants. Describe methods of follow-up<br>(*b*) For matched studies, give matching criteria and number of exposed and unexposed	3
Variables	7	Clearly define all outcomes, exposures, predictors, potential confounders, and effect modifiers. Give diagnostic criteria, if applicable	4-5, Table 1
Data sources/ measurement	8*	For each variable of interest, give sources of data and details of methods of assessment (measurement). Describe comparability of assessment methods if there is more than one group	4-5, Table 1
Bias	9	Describe any efforts to address potential sources of bias	6
Study size	10	Explain how the study size was arrived at	-
Quantitative variables	11	Explain how quantitative variables were handled in the analyses. If applicable, describe which groupings were chosen and why	5-6
Statistical methods	12	(*a*) Describe all statistical methods, including those used to control for confounding<br>(*b*) Describe any methods used to examine subgroups and interactions<br>(*c*) Explain how missing data were addressed<br>(*d*) If applicable, explain how loss to follow-up was addressed<br>(*e*) Describe any sensitivity analyses	5-6
**Results**			
Participants	13*	(a) Report numbers of individuals at each stage of study—eg numbers potentially eligible, examined for eligibility, confirmed eligible, included in the study, completing follow-up, and analysed<br>(b) Give reasons for non-participation at each stage<br>(c) Consider use of a flow diagram	6, Figure 1
Descriptive data	14*	(a) Give characteristics of study participants (eg, demographic, clinical, social) and information on exposures and potential confounders	6-8, Table 2

		(b) Indicate number of participants with missing data for each variable of interest<br>(c) Summarise follow-up time (eg, average and total amount)	
Outcome data	15*	Report numbers of outcome events or summary measures over time	8-9, Table 3
Main results	16	(*a*) Give unadjusted estimates and, if applicable, confounder-adjusted estimates and their precision (eg, 95% confidence interval). Make clear which confounders were adjusted for and why they were included<br>(*b*) Report category boundaries when continuous variables were categorized<br>(*c*) If relevant, consider translating estimates of relative risk into absolute risk for a meaningful time period	8-9, Table 3
Other analyses	17	Report other analyses done—eg analyses of subgroups and interactions, and sensitivity analyses	-
**Discussion**			
Key results	18	Summarise key results with reference to study objectives	10
Limitations	19	Discuss limitations of the study, taking into account sources of potential bias or imprecision. Discuss both direction and magnitude of any potential bias	11
Interpretation	20	Give a cautious overall interpretation of results considering objectives, limitations, multiplicity of analyses, results from similar studies, and other relevant evidence	10-11
Generalisability	21	Discuss the generalisability (external validity) of the study results	10
**Other information**			
Funding	22	Give the source of funding and the role of the funders for the present study and, if applicable, for the original study on which the present article is based	11

*Give information separately for exposed and unexposed groups.

Note: An Explanation and Elaboration article discusses each checklist item and gives methodological background and published examples of transparent reporting. The STROBE checklist is best used in conjunction with this article (freely available on the Web sites of PLoS Medicine at http://www.plosmedicine.org/, Annals of Internal Medicine at http://www.annals.org/, and Epidemiology at http://www.epidem.com/). Information on the STROBE Initiative is available at http://www.strobe-statem

Table S2. Details of the intervention protocol.

Intervention Component	Main goals	Description
**Education**		
Initial preparation before PFMT	Introduction to the basic technical aspects needed before initiating PFMT<br><br>Improve pelvic floor muscles awareness	Comprised the following topics:<br>Basic anatomy and physiology of the pelvic floor<br>The role and importance of exercise<br>How to contract and relax the PFM<br>Diaphragmatic breathing (which was adopted during PFMT)<br>How to insert, use, and sanitize the intravaginal sensor<br><br>These aspects were introduced in the initial preparation before PFMT.
Education about pelvic health and specific for the condition	Improve patient's literacy on pelvic health and the condition<br><br>Facilitate the adoption of healthy lifestyle habits<br><br>Empowering patient's self-management of the condition	Comprised the following topics:<br>Pelvic floor: anatomy and functions<br>Menopause symptoms<br>Pathophysiology of the pelvic condition<br>Contributing factors for the pelvic condition<br>Mental health<br>Relaxation techniques<br>Lifestyle modification advice and strategies<br>Healthy toileting habits<br><br>These aspects were introduced after the completion of the initial preparation until the program-end.
**Pelvic floor muscle training**		
PFMT isolated and PFMT in combination with functional exercises	Improve pelvic floor power, endurance, speed, and adaptability<br><br>Improve coordination between the PFM and surrounding muscle groups (e.g., abdominal muscles, hip muscles)<br><br>Improve pelvic girdle and pelvic floor mobility	PFMT comprised four categories of exercise:<br>1) Strength, requiring a maximum contraction of PFM;<br>2) Endurance, requiring a sustained contraction of the PFM at a target level;<br>3) Control, requiring contractions and relaxations at a target level;<br>4) Agility, requiring rapid contractions followed by a relaxation at a target level to simulate the activity of the PFM in reflex contractions, such as during coughing.<br><br>PFMT was initiated after the initial education.<br><br>PFMT was combined with strengthening functional exercises (including abdominal and lower limb exercises)

	After the completion of ≥3 sessions, PFMT gradually progressed through the combination of PFMT with functional exercises until the program-end.<br><br>Example of a session:<br>1. Strength for 3 reps<br>2. Control - Contraction 6:4 seconds on/off for 59 seconds (116 targets*)<br>3. Hold - Contraction 2:2 seconds on/off for 3 reps<br>4. Agility - Contraction for 10 reps every 1 second<br>5. Bridge with Pelvic Floor Contractions for 5 reps<br>6. Hold - Contraction 3:3 seconds on/off for 3 reps<br>7. Diaphragmatic Breathing for 10 reps

Note: Exercise sessions were tailored by the physical therapist according to participant’s condition and progression, namely the number of repetitions and sets, and the duration of contractions and relaxations.
*The target level refers to the degree of contraction or relaxation for a given exercise.

Table S3. Model fit estimates for the unconditional model: intention-to-treat approach.

Outcome	Model fit				
	**Chi-sq (df)**	**P-value**	**RMSEA**	**CFI**	**SRMR**
PFIQ-7	1.126 (1)	***0.29***	**0.006**	**1.00**	**0.005**
UIQ-7	1.733 (1)	***0.19***	**0.016**	**1.00**	**0.007**
CRAIQ-7	0.594 (1)	***0.44***	**0.000**	**1.00**	**0.004**
POPIQ-7	1.668 (1)	***0.20***	**0.015**	**1.00**	**0.007**
Pelvic Health Symptoms >0	15.852 (3)	***0.001***	**0.039**	**0.99**	**0.048**
WPAI Overall>0	12.085 (6)	***0.06***	**0.031**	**0.98**	**0.038**
WPAI Work >0	1.217 (6)	***0.98***	**0.000**	**1.00**	**0.024**
WPAI Activities >0	12.309 (6)	***0.05***	**0.026**	**0.99**	**0.039**

Abbreviations: CRAIQ-7 Colo-Rectal-Anal Impact Questionnaire - short form 7; PFIQ-7, Pelvic Floor Impact Questionnaire - short form 7; POPIQ-7, Pelvic Organ Prolapse Impact Questionnaire - short form 7; UIQ-7, Urinary Impact Questionnaire - short form 7; WPAI, Work Productivity and Activity Impairment Questionnaire.

Note: Model fitness was assessed through chi-squared test, root mean square error of approximation (RMSEA), confirmatory fit index (CFI), and standardized root mean square residual (SRMR), according to the criteria: CFI = close to 0.95; RMSEA = close to 0.06 and SRMR = close to 0.08. Significant p-values and model fit values indicating good model fit are presented in bold.

Table S4. Conditional model with age, body mass index (BMI), hormone replacement therapy, SDI and geographical location as covariates.

Outcomes:		Age		BMI		Hormone Replacement Therapy (yes)		SDI		Rurality (rural)	
		**Mean (95% CI)**	**P-value**	**Mean (95% CI)**	**P-value**	**Mean (95% CI)**	**P-value**	**Mean (95% CI)**	**P-value**	**Mean (95% CI)**	**P-value**
**PFIQ-7**	Intercept	-1.43 (-3.27;0.41)	*.13*	4.97 (3.10;6.83)	***<.001***	-3.00 (-9.56;3.57)	*.37*	3.72 (1.85;5.60)	***<.001***	-1.69 (-6.97;3.59)	*.53*
	Slope	0.13 (-0.19;0.46)	*.42*	0.23 (-0.11;0.58)	*.18*	-0.54 (-1.65;0.56)	*.34*	-0.12 (-0.45;0.21)	*.47*	0.70 (-0.22;1.61)	*.13*
	Curve	0.00 (-0.02;0.01)	***.007***	-0.02 (0.04;-0.01)	***.007***	0.03 (-0.03;0.08)	*.31*	0.00 (-0.01;0.02)	*.67*	-0.04 (-0.09;0.00)	*.06*
**UIQ-7**	Intercept	-0.48 (-1.29;0.33)	*.24*	2.86 (2.05;3.68)	***<.001***	-2.03 (-4.91;0.85)	.17	1.57 (0.75;2.39)	***<.001***	-1.18 (-3.49;1.13)	*.32*
	Slope	0.04 (-0.10;0.17)	*.60*	0.07 (-0.07;0.22)	*.34*	-0.05 (-0.51;0.41)	.84	-0.08 (-0.21;0.06)	*.29*	0.36 (-0.02;0.75)	*.06*
	Curve	0.00 (-0.01;0.01)	*.75*	-0.01 (-0.02;0.00)	***.017***	0.01 (-0.02;0.03)	.63	0.00 (0.00;0.01)	*.39*	-0.02 (-0.04;0.00)	***.027***
**CRAIQ-7**	Intercept	0.07 (-0.66;0.79)	*.86*	1.23 (0.48;1.97)	***.001***	-0.97 (-3.53;1.60)	.46	1.24 (0.50;1.97)	***.001***	-0.43 (-2.51;1.66)	*.69*
	Slope	0.01 (-0.12;0.14)	*.87*	0.06 (-0.07;0.20)	*.36*	-0.23 (-0.66;0.21)	.30	-0.10 (-0.24;0.03)	*.12*	0.32 (-0.04;0.68)	*.08*
	Curve	0.00 (-0.01;0.01)	*.86*	-0.01 (-0.01;0.00)	*.05*	0.01 (-0.01;0.03)	.32	0.00 (0.00;0.01)	*.28*	-0.02 (-0.04;0.00)	***.048***
**POPIQ-7**	Intercept	-1.13 (-1.87;-0.39)	***.003***	1.09 (0.34;1.84)	***.004***	-0.35 (-2.96;2.26)	.79	1.01 (0.25;1.76)	***.009***	-0.23 (-2.34;1.88)	*.83*
	Slope	0.07 (-0.07;0.21)	*.31*	0.13 (-0.02;0.28)	*.09*	-0.27 (-0.73;0.20)	.26	0.05 (-0.09;0.19)	*.51*	0.08 (-0.31;0.46)	*.69*
	Curve	0.00 (-0.01;0.00)	*.51*	-0.01 (-0.02;0.00)	***.008***	0.01 (-0.01;0.03)	.30	0.00 (-0.01;0.00)	*.46*	-0.01 (-0.03;0.01)	*.33*
**Pelvic Health Symptoms >0**	Intercept	0.22 (0.14;0.29)	***<.001***	0.08 (0.00;0.15)	***.047***	-0.06 (-0.32;0.20)	.64	0.09 (0.02;0.17)	***.013***	-0.13 (-0.35;0.08)	*.21*
	Slope	-0.03 (-0.14; 0.09)	*.64*	0.12 (0.00; 0.25)	***.044***	0.02 (-0.37; 0.41)	.93	-0.14 (-0.26; -0.03)	***.016***	0.32 (0.00; 0.64)	*.05*
**WPAI Overall >0**	Intercept	-0.58 (-2.04;0.88)	*.44*	0.68 (-0.66;2.02)	*.32*	2.89 (-1.97;7.75)	.24	0.95 (-0.44;2.34)	*.18*	-3.08 (-6.95;0.78)	*.12*
	Slope	0.78 (-1.08;2.64)	*.41*	0.95 (-0.91;2.82)	*.32*	-3.06 (-9.12;2.99)	.32	0.99 (-0.84;2.83)	*.29*	-0.54 (-5.43;4.35)	*.83*

**WPAI Work >0**	Intercept	-1.04 (-2.32;0.25)	*.11*	0.88 (-0.29;2.05)	*.14*	1.51 (-2.69;5.72)	.48	0.50 (-0.72;1.72)	*.42*	-2.64 (-5.98;0.71)	*.12*
	Slope	0.81 (-0.88;2.50)	*.35*	1.13 (-0.56;2.81)	*.19*	-0.23 (-5.61;5.15)	.93	1.10 (-0.57;2.77)	*.20*	-0.06 (-4.43;4.31)	*.98*
**WPAI Activity >0**	Intercept	-1.57 (-2.69;-0.46)	***.006***	1.63 (0.56;2.70)	***.003***	-3.53 (-7.43;0.37)	.08	0.51 (-0.60;1.62)	*.37*	-3.13 (-6.20;-0.06)	***.045***
	Slope	1.01 (-0.43;2.44)	*.17*	0.11 (-1.34;1.57)	*.88*	1.65 (-3.18;6.48)	.50	1.24 (-0.20;2.69)	*.09*	0.64 (-3.27;4.55)	*.75*

Abbreviations: BMI, body mass index; CRAIQ-7 Colo-Rectal-Anal Impact Questionnaire short form 7; PFIQ-7, Pelvic Floor Impact Questionnaire - short form 7; POPIQ-7, Pelvic Organ Prolapse Impact Questionnaire short form 7; SDI, Social Deprivation Index; UIQ-7, Urinary Impact Questionnaire - short form 7; WPAI, Work Productivity and Activity Impairment Questionnaire.

Note: The effect of covariates on the curve estimate was not calculated for Pelvic Health Symptoms and WPAI outcomes, as these were assessed through latent-basis growth analysis (LBGA). Significant p-values are presented in bold.

Table S5. Model fit estimates for the conditional model.

**Outcome**	**Model fit**				
	**Chi-sq (df)**	***P-value***	**RMSEA**	**CFI**	**SRMR**
PFIQ-7	1.597 (6)	***0.95***	**0.000**	**1.00**	**0.003**
UIQ-7	5.703 (6)	***0.46***	**0.000**	**1.00**	**0.006**
CRAIQ-7	1.354 (6)	***0.97***	**0.000**	**1.00**	**0.003**
POPIQ-7	3.663 (6)	***0.72***	**0.000**	**1.00**	**0.006**
Pelvic Health Symptoms >0	31.743 (13)	***0.003***	**0.022**	**0.99**	**0.029**
WPAI Overall >0	24.808 (16)	***0.07***	**0.023**	**0.98**	**0.029**
WPAI Work >0	13.406 (16)	***0.64***	**0.000**	**1.00**	**0.022**
WPAI Activities >0	19.166 (16)	***0.26***	**0.011**	**1.00**	**0.023**

Abbreviations: CRAIQ-7 Colo-Rectal-Anal Impact Questionnaire - short form 7; PFIQ-7, Pelvic Floor Impact Questionnaire - short form 7; POPIQ-7, Pelvic Organ Prolapse Impact Questionnaire - short form 7; UIQ-7, Urinary Impact Questionnaire - short form 7; WPAI, Work Productivity and Activity Impairment Questionnaire.

Note: Model fitness was assessed through chi-squared test, root mean square error of approximation (RMSEA), confirmatory fit index (CFI), and standardized root mean square residual (SRMR), according to the criteria: CFI = close to 0.95; RMSEA = close to 0.06 and SRMR = close to 0.08. Model fit values indicating good model fit are presented in bold.
